# Bottlenecks to HIV care and treatment in sub-Saharan Africa: a multi-country qualitative study

**DOI:** 10.1136/sextrans-2017-053172

**Published:** 2017-07-23

**Authors:** Alison Wringe, Jenny Renju, Janet Seeley, Mosa Moshabela, Morten Skovdal

**Affiliations:** 1 Department of Population Health, London School of Hygiene and Tropical Medicine, London, UK; 2 Medical Research Council/Uganda Virus Research Institute Research Unit on AIDS, Entebbe, Uganda; 7 Department of Global Health, London School of Hygiene and Tropical Medicine, London, UK; 3 Africa Health Research Institute, KwaZulu Natal, South Africa; 4 University of KwaZulu Natal, Durban, South Africa; 5 University of Copenhagen, Copenhagen, Denmark; 6 Biomedical Research and Training Institute, Harare, Zimbabwe

**Keywords:** HIV, sub-Saharan Africa, antiretroviral therapy, engagement, health services

The expansion in the provision of life-saving antiretroviral therapy (ART) in sub-Saharan Africa over the past 15 years has been an unprecedented achievement for public health. By the end of 2015, an estimated 10.3 million persons living with HIV (PLHIV) were receiving ART in southern and eastern Africa, the most affected region in the world. Just over half of all PLHIV in the region are now receiving ART, more than double the number just 3 years earlier.^1^

ART scale-up has dramatically reduced HIV-related mortality and morbidity, bringing countless social and economic benefits to communities that had been hard hit by the epidemic. The network for Analysing Longitudinal Population data on HIV/AIDS (ALPHA), a collaboration among 10 health and demographic surveillance **system (HDSS)** sites in east and southern Africa, has been investigating declines in adult mortality and their causes in seven countries (www.lshtm.ac.uk/alpha).^2^ Recent analyses from seven sites indicate a substantial impact of HIV treatment programmes on adult mortality following the expansion of ART, with declines ranging from 58% to 84%.^3^

Despite this progress, there remains a substantial deficit in overall life expectancy among adults living with HIV, with their survival between 5 and 10 years less than among uninfected adults.^4^ These ‘excess’ deaths among PLHIV are occurring due to late diagnosis, poor linkage to care and treatment, and ART interruptions.^5^ By the end of 2015, an estimated 44% of PLHIV in southern and eastern Africa remained undiagnosed,^6^ and rates of linkage to care after diagnosis range widely from 17% to 78%, while ART initiation among those eligible for treatment ranges from 14% to 95%.^7^ A number of studies have documented reasons for delays in testing, and initiating and adhering to treatment including beliefs that treatment is for people who are sick, fear of side effects and concerns about stigma.^8–11^

In 2014, the Joint United Nations Programme on HIV and AIDS and partners set the ‘90-90-90 targets’ to drive the efforts for AIDS elimination by 2030, prompting the widespread adoption of ‘test and treat’ strategies. Understanding and addressing these existing bottlenecks in HIV care and treatment will be critical to attaining the 90-90-90 targets and achieving a future free of AIDS. Qualitative research can help to elucidate the social norms, practices and interactions that drive patients’ varied engagement with HIV care and treatment services in different contexts. By exploring the perspectives of HIV service users, their family member and health providers, it is possible to elicit how the interactions between patients and health services take form and are shaped by broader community responses to the epidemic.

In this context, we undertook a multicountry qualitative study to explore the bottlenecks to HIV care and treatment in seven HDSS sites  within the ALPHA Network: Kisumu (Kenya), Rakai, Kyambaliwa (Uganda), Kisesa (Tanzania), Karonga (Malawi), uMkhanyakude (South Africa) and Manicaland (Zimbabwe). The overall aim of the ‘Bottlenecks Study’ was to explore how contextual, social and health systems factors influence the engagement of PLHIV with HIV care and treatment in seven HDSS in sub-Saharan Africa. By undertaking the research in health and demographic surveillance sites, we were able to sample PLHIV with different HIV care and treatment histories, including those who had been diagnosed but not yet initiated care, or those who had disengaged from the HIV care process. Both of these groups are notoriously difficult to include in qualitative studies, partly due to difficulties in recruiting them in the absence of household identifiers and preobtained consent to contact them for research purposes. As a result, their voices and stories are often missing from accounts of HIV service use, which can risk focusing policy recommendations on those who face relatively fewer challenges to HIV care engagement. Furthermore, the availability of verbal autopsy data sets in HDSS sites, used to assign cause of death for better HIV surveillance, enabled us to contact and interview the family members of some PLHIV who had died from AIDS-related causes, providing a rarely obtained family member’s perspective of the events and circumstances surrounding the death of their relative. Further details on the sampling frames, study settings and methods for the study in each site are available as online Supplementary file 1.

10.1136/sextrans-2017-053172.supp1 Supplementary material

Undertaking a qualitative study across so many settings presents a multitude of challenges, as well as opportunities, not least in relation to the analysis and interpretation of the data. In our study, all of the sites had some characteristics in common, namely their rural nature, similar provision of HIV treatment services through small health centres and dispensaries since 2004–2005, and a generalised HIV epidemic. Despite these similarities, drawing out comparisons between study sites in qualitative studies can be complex, because some apparent differences may reflect how the data were produced, rather than variation in the implementation of national HIV policies or sociocultural contexts, with the former being more complex to attribute to the observed differences in the data. For these reasons, for each paper, we chose to include data from sites where the findings were broadly similar.

In this special issue of *Sexually Transmitted Infections*, we present the findings from the Bottlenecks Study. We chose to explore patients’ experiences at different stages of HIV care and treatment, that is, HIV testing, access to ART and retention, with a view to understanding how PLHIV, in the context of their social worlds, interact with these HIV services. Combined, the papers provide an analytical spotlight to the multiple tensions that exist between HIV programmes and the complex social realities that often characterise the lives of PLHIV in each study setting.

In a guest editorial to this special issue, Kielmann and Cataldo argue that bottlenecks to HIV care and treatment can be situated historically in the context of previous ART roll-out approaches. They explore how spatial, temporal and relational parameters of the current drive towards universal test-and-treat models have evolved over the past 15 years.^12^

In the first research article in this special issue, Wringe and colleagues consider how experiences of PLHIV during their HIV testing encounters in the different HDSS may influence subsequent engagement in HIV care.^13^ They show that some of the principles that should underlie all HIV testing and counselling practices, such as consent, confidentiality and good quality counselling, may be modified or omitted by health workers to achieve perceived health benefits or policy targets. They argue that while such actions arguably save lives and increase HIV diagnosis rates, they may also jeopardise efforts to connect many diagnosed PLHIV to the long-term HIV care necessary for elimination of AIDS by 2030.

Bukenya and colleagues^14^ focus their research on those PLHIV who, despite knowing their HIV status, have not yet initiated HIV treatment. In trying to understand why this is the case, they unravel a range of persistent barriers pertaining to the affordability, availability and acceptability of HIV services. The barriers suggest that current models of HIV care and delivery still fail to consider the sociocultural context and economic position of PLHIV. The authors call for greater patient-centredness in the delivery of HIV care and treatment.

In their paper, Renju and colleagues pick up on an observation also made by Bukenya *et al*, namely that embodied experiences of HIV influence their acceptance of ART.^15^ The authors also note that across all the study sites, health providers, in an effort to achieve viral suppression, often failed to recognise the immediate needs that PLHIV face when enduring debilitating side effects. This could contribute to disengagement with HIV services, particularly among PLHIV who had not experienced the transformational effects of ART. They hypothesise that PLHIV who have not experienced AIDS-defining illnesses are less likely to find the motivation for tolerating incapacitating side effects that are common with ART.

The study by McLean and colleagues on Option B+ for the prevention of mother-to-child transmission services in Malawi, Tanzania and Uganda highlights the importance of identifying and capitalising on factors that motivate engagement with HIV care and treatment attenuating the impact of potential barriers.^16^ They found that a woman’s role as a mother was a powerful motivator to accept HIV care and treatment, over-riding other barriers to starting ART. Healthcare workers used the narrative of ‘protecting the unborn child’ tactically to amplify the benefits of this motivator.

Bonnington and colleagues examine the role of stigma in shaping engagement with HIV services in all of the study sites. By looking at the temporality of stigma (stigma in the everyday, over biographical time or historically), the authors disentangle different manifestations of stigma.^17^ They highlight how stigmatising attitudes, in their multiple and temporal forms, often disrupt testing and can also contribute to the termination of postdiagnosis care. They call for stigma mitigation strategies that consider the temporality of stigma, tailored to the different stages of HIV care.

The interactions between patients and providers throughout the spectrum of HIV care and treatment services also influence patient engagement in care. Ondenge and colleagues elicit some of the broader contextual factors and dynamics that shape patient–provider interactions in five of the study sites.^18^ Their analysis demonstrates that patient–provider interactions are complex, multidimensional, deeply embedded in wider social dynamics and shaped by multiple ecological domains. They argue that greater efforts are needed to enhance patient–provider dialogue and empower patients to make decisions about their own health to improve their engagement in HIV care.

National HIV programmes are not the only providers of HIV care. Moshabela and colleagues look at the multitude of actors engaged with HIV care and examine how biomedical HIV services intersect with traditional, complementary and alternative medicine and care in all the sites.^19^ They find that although traditional/faith-based care was mostly treated as secondary and complementary to biomedical treatment, medical pluralism mediated pathways to HIV care continuum through treatment delays and interruptions and further fuelled fears of drug-to-drug interactions. The authors call for more collaborative culture-sensitive approaches to healthcare in sub-Saharan Africa.

Wamoyi and colleagues draw our attention to the interplay between couple dynamics and HIV care-seeking behaviour in South Africa, Tanzania and Malawi.^20^ Their analysis reveals that couple dynamics influenced engagement with HIV testing, care and treatment for both partners through a myriad of pathways. Supportive partnerships could strengthen engagement with HIV services; however, for many couples, fear of blame, abandonment or abuse resulted in unwillingness to disclose HIV status to each other. Many couples therefore did not engage with HIV services together, and instead allowed secrecy and mistrust to interfere with their HIV treatment and care.

By way of summarising, Skovdal and colleagues consider the potential of ‘theories of practice’ for studying and understanding these varied engagements with HIV services across all of the participating sites.^21^ Their theoretical bird’s-eye perspective suggests that engagement with HIV services is contingent on the availability, absence and connections between requisite ‘materialities’ (eg, health infrastructure, medicines), ‘competencies’ (eg, knowing what and knowing how to live with HIV) and ‘meanings’ (eg, trust in HIV services, stigma, normalisation of HIV) and other life practices.

In conclusion, the papers in this volume illustrate how the ability of PLHIV to engage with HIV services is limited by a range of relational, symbolic, material and institutional factors in study settings across eastern and southern Africa. Greater acknowledgement of these issues, and imaginative, bold approaches to address them, must be at the forefront of policies and programmes to deliver HIV care and treatment, if the ambitious goal of AIDS elimination by 2030 is to be reached.
