# Supplementary material for: Bottlenecks to HIV care and treatment in sub-Saharan Africa: a multi-country qualitative study
Source: Sex Transm Infect. 2017 Jul 23;93(Suppl 3):e053172. doi: 10.1136/sextrans-2017-053172 (PMC5739850; doi:10.1136/sextrans-2017-053172)
Supplement: Supplementary material [file sextrans-2017-053172supp001.pdf]

The HIV Bottlenecks study methodology

The HIV Bottlenecks study was a multisite qualitative study nested within seven health and demographic surveillance sites in six countries in sub-Saharan Africa. The study aimed to explore engagement of people living with HIV (PLHIV) with HIV care, and treatment services being offered through the national HIV programmes.

Each of the papers in this special issue either provides an indepth exploration of how and why bottlenecks occur at different stages of the HIV care and treatment process (including HIV testing, accessing antiretroviral therapy (ART) and retention in care) or examines how recurrent structural and social factors such as stigma, gender inequities, medical pluralism or patient–provider relationships contribute to the development of these bottlenecks. Each article includes a brief description of the key methods that are relevant to that particular paper. This supplement is intended to accompany each of the papers in this series in order to provide more comprehensive information on the methods used in the Bottlenecks study overall and by each site. We begin by giving an overview of the study sites, and then describe the sampling strategies and data collection methods that were adopted in each site. We also describe the data-management strategies and methods of analyses that were undertaken by each study site.

STUDY SITES

The study was set within seven health and demographic surveillance sites that were members of the Alpha network (http://alpha.lshtm.ac.uk/). These sites were selected as those already contributing to a parallel study being conducted to estimate mortality across the HIV care cascade and the rates of transition between cascade stages. These were: Karonga (Malawi), Kisesa (Tanzania), Kisumu (Kenya), Manicaland (Zimbabwe), Kyamulibwa and Rakai (Uganda), and uMkhanyakude (South Africa) (see table 1). Detailed descriptions of each study site have been published elsewhere [1–3] and are summarised in table 1.

By conducting the research within HDSS, we were able to sample PLHIV at different stages of the HIV care and treatment cascade, including those who were lost to follow-up from care as they were still included in the demographic surveillance rounds. Similarly, we were also able to use information from verbal autopsy (VA) interviews conducted with family members of persons who had recently died from HIV, and whose HIV status was known to their family.

PROTOCOL AND TOOL PREPARATION

A week-long workshop was convened in London, attended by the lead social scientist for the study from each site. The group worked together to discuss and refine the different sampling groups and the various strategies that could be adopted to reach these groups and obtain samples. A generic protocol was developed in this workshop. Each study site was then responsible for modifying the generic protocol and developing site-specific protocols which would guide their study. These protocols were then submitted to national and institutional ethical review boards in the partner countries.

During this workshop, generic topic guides were developed based on previous research in order to explore particular issues of interest. Study coordinators from each site then developed these guides further to include any additional areas of interest that were specific to their setting.

THE SAMPLING STRATEGY

Sampling for PLHIV

The sampling approach was designed to ensure that we could identify PLHIV in relation to their interactions with the HIV care and treatment programme (see figure 1), and thus capture a diverse range of examples of how contextual and programmatic issues shape their care-seeking behaviour. Specifically, individuals were sampled according to four broad criteria, as described below. In addition, the sampling was conducted to include 'deviant' or 'extreme' cases within each category, as well as covering a broad range of ages and both sexes.

Sites sampled participants across variables that may influence the experiences of PLHIV in accessing HIV services in their setting, such as their pregnancy status, area of residence or livelihood. For example, pregnant women accessing option B+ were purposively sampled in Kyamulibwa, Kisesa and Karonga, while fishermen and commercial sex workers were purposively sampled in Rakai and Manicaland, respectively. Table 2 shows the number of participants by sampling category.

The four broad sampling groups were:

1. *PLHIV diagnosed but not initiated on ART*

Understanding the experiences of PLHIV who had been diagnosed with HIV, but had not yet initiated treatment was critical to achieving the study's overall aims.[5] People in this group are notoriously difficult to recruit into such studies as in most cases they are not regularly attending pre-ART care services.

The sampling for this group either targeted individuals according to their ART eligibility status: eligible for ART, not yet eligible for ART (determined according to CD4 level or clinical stage), or had never undergone an eligibility assessment; or according to whether they were in contact with an HIV care and treatment clinic (regularly and sporadically) or not. The target was to include approximately 10 individuals in this category: five eligible for ART and five not yet eligible, or five persons attending pre-ART care and five who were currently not attending pre-ART care (lost to clinic

Table 1 Key characteristics of the Bottlenecks study sites[4]

Country	HDSS site	Size of HDSS site (km^2)	Approximate population of HDSS site	HIV prevalence (%)
Kenya	Kisumu (KEMRI/CDC)	369	142 000	15
Uganda	Rakai	320	32 000	13
Uganda	Kyamulibwa	54.3	21 000	9
Tanzania	Kisesa	150	30 000	7
Malawi	Karonga	135	39 000	7
Zimbabwe	Manicaland	36 459	11 000	15
South Africa	uMkhanyakude	438	90 000	33

CDC, Centers for Disease Control and Prevention; HDSS, Health and Demographic Surveillance System; KEMI, Kenya Medical Research Institute.

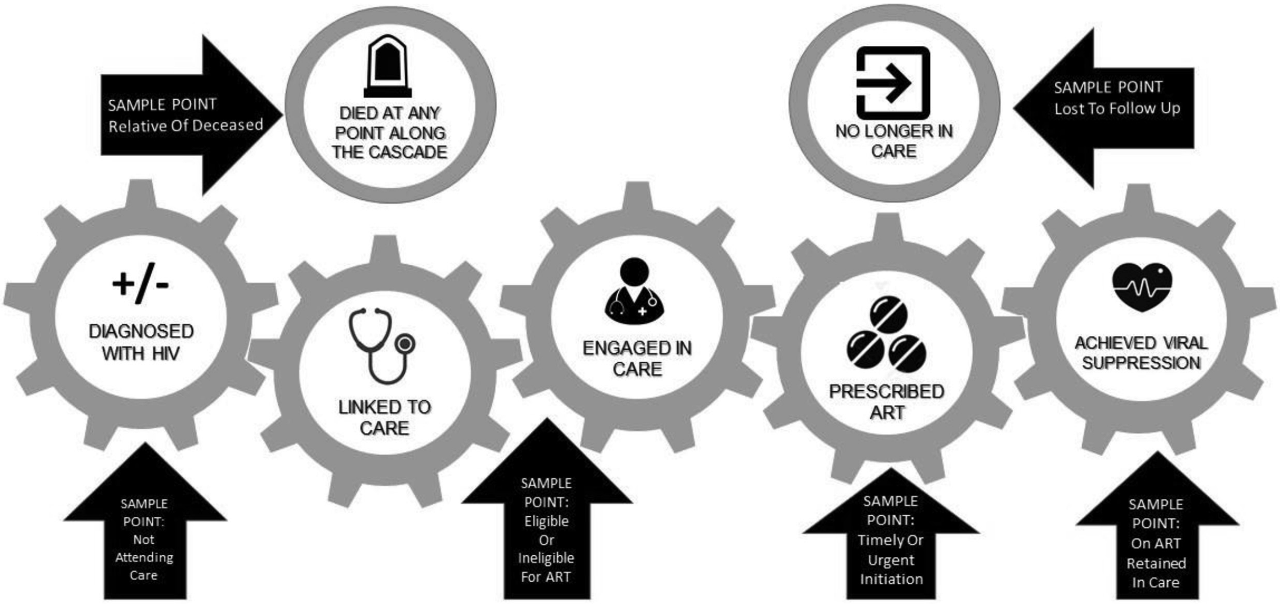

Figure 1 Schematic illustrating the different sampling points along the HIV care and treatment continuum that was adopted in the HIV Bottlenecks study. (Modified from human & resource services administration (https://hab.hrsa.gov/about-ryan-white-hivaids-program/hiv-care-continuum) ART, antiretroviral therapy.

Table 2 Participant by treatment history and country

	Target	Kyamulibwa	Karonga	Kisesa	Rakai	Manicaland	uMkhanyakude	Kisumu	Total
Diagnosed but not on ART*	10	8	9	13	15	16	16	10	87
On ART†	10	16	27	20	15	35	17	15	145
Lost to follow-up‡	5	4	4	4	6	8	6	6	38
Recently died of AIDS§	5	5	6	6	8	6	6	11	48
Healthcare worker¶	5	5	5	7	6	4	19	8	54
Grand total	35	38	51	50	50	69	64	50	365
Refusals		2	0	1	0	4	0	0	7
Repeat interviews		0	5	1	0	0	0	0	6

Participants included both men and women of varying ages.
*Subcategories included those who (according to current guidelines) were either eligible or not yet eligible for ART. Participants in this group may or may not have been in contact with HIV care and treatment services.
†The PLHIV could have been taking ART for variable periods of time, countries had varying cut-off points but generally captured, recently initiated and then longer term (over 5 years).
‡PLHIV had not collected ART from their registered clinic for a country-specific predetermined period of time.
§Relatives of those who had been diagnosed and had disclosed their HIV status and had recently died were identified and interviewed.
¶Representing various different cadres.
ART, antiretroviral therapy; PLHIV, people living with HIV.

follow-up during the pre-ART phase or never enrolled at an HIV clinic following an HIV diagnosis).

This group was recruited using various approaches which depended on their current history of contact with the ART programme, programme implementation (ie, presence of home-based care programmes, post-test clubs, HDSS and HIV treatment databases, etc) and related ethical considerations that were specific to each site. Some sites with recently updated linked HDSS and HIV clinic datasets could identify potential participants through this dataset and stratify participants according to their current history of contact with the ART programme following diagnosis. The 'seeded focus group' approach[6] was also used in one site (Kisesa), in which community members, including PLHIV (identified through the HDSS dataset or by HCT counsellors) were invited to participate. Individuals of interest were then invited to attend an interview on a subsequent date at the end of the group session. In all sites PLHIV who were in contact with care were recruited from pre-ART care. In other sites, those who were not yet enrolled in pre-ART care were recruited through local community organisations or outreach programmes (eg, home-based care programmes, post-test PLHIV clubs, etc), and through HCT counsellors who had knowledge of those diagnosed within the community. Additionally, in one site (Manicaland), a snowballing approach was adopted in which a study participant would invite his/her friend to take part in the study; this was most appropriate for commercial sex workers.

Country research teams carefully considered the most appropriate way to identify these PLHIV to ensure that ethical standards were met.

2. *Recently initiated on ART*

The rationale for interviewing individuals who had recently initiated ART was driven by the fact that in all sites, a large proportion of deaths among HIV-positive persons occur in individuals within 6 months of treatment initiation.[7]

The sampling for this group included individuals who

a. initiated ART in a 'timely' way—ie, those who were in regular pre-ART care until they reached the local ART eligibility criteria, and/or those initiating ART with CD4 counts at the higher end of the threshold;
b. were urgently initiated on treatment following an eligibility assessment, including individuals who were referred from/currently in hospital or health facility inpatient departments, tuberculosis wards or those in poor health and with low CD4 counts documented during outpatient care.

PLHIV were recruited from clinics within the HDSS catchment area, and recruited following ART initiation consultations. Screening was undertaken to determine that patients had characteristics of interest for the study and/or were members of the HDSS. Arrangements were made to interview in venues preferred by the participant, which were most usually at or near the clinic or at participant's homes, and within nearby school structures, within 1 or 2 weeks of initiation, usually when the patient attended a planned follow-up appointment. Most sites used the pre-ART and ART registers in clinics within the HDSS as a sampling frame to identify potential participants for timely initiators. This enabled us to know how long they had been in care. One site recruited patients who were attending their first appointments directly from the clinics. For the urgent initiators, some sites used CD4 count records or clinical records within these clinics were used for identification and enrolment, others classified urgent initiators as those who had been hospitalised recently for AIDS-related illness or had not been attending their pre-ART appointments. The target was to include approximately 10 individuals, with around half representing 'urgent' initiators, and the other half 'timely' initiators per site.

Repeat interviews were conducted with a few individuals within this group to understand the changes that they experienced during this early phase of ART. The second interviews were planned for approximately 2 months following initiation, to allow respondents time to adapt to treatment or recover from opportunistic infections.

3. *Initiated on ART but no-longer in care: lost to follow-up*

These individuals represented an important group within the sampling frame, being at high risk of death but still alive. They were also the most challenging to recruit, because of the factors which resulted in them no longer being in care. The sampling frame for these individuals used the linked HDSS and clinic datasets in some settings. In other sites, clinic-based lists of individuals who were lost to follow-up were used as a starting point, and the linked HDSS and clinic data were used to determine the place of residence of the individual within the study area. In one site, only clinic-based records were used to identify these participants. The minimum sample size for this group was five PLHIV. Oversampling was conducted in light of the challenges to find these PLHIV and due to risk that some may have recently died.

Sites had to adopt their own innovative strategies to invite PLHIV in this category to participate in the study: some used clinic-based tracing nurses/home-based care workers while others used case managers. All those involved in recruitment had already obtained permission from the patients for household visits to be made in the event of missed appointments.

This group included individuals who had defaulted from treatment for different periods of time, including recent defaulters who had not attended a scheduled appointment. The definition of 'lost to follow-up' varied slightly by site but was typically three months.[8]

4. *Currently on ART, and initiated at least 6 months earlier*

Individuals falling within this category had first initiated ART at least 6 months earlier so that they were no longer in the group at highest risk of mortality. This category included PLHIV who were 'clinically stable' and had regularly attended scheduled appointments as well as those who had been on ART for at least 6 months with irregular patterns of attendance, but were not considered as currently lost to follow-up.

In some sites, clinic records were used to identify patients who had initiated ART more than 6 months previously. Other sites used the ART clinic records to identify patients on ART for more than 6 months and then used the linked HDSS information and records to ensure residency within the HDSS. Patients' reports were then used to determine the regularity of individuals' clinic attendance over the past 6 months, the number of months that had elapsed since starting ART and an indication of their clinical state. The target number for this group was five PLHIV on ART.

5. *Family members of deceased persons living with HIV and in contact with HIV services*

Exploring the underlying circumstances surrounding deaths of PLHIV was critical to achieving a full understanding of barriers to care-seeking in these communities. Those who had died were typically individuals who were the least successful in terms of achieving timely and sustained engagement with HIV services.

Interviews were therefore also conducted with selected family members of PLHIV who had died in the past five years (chosen to correspond with increasing ART availability and HIV care accessibility). All study sites regularly conduct VAs with families of any deceased person in the area, as identified through the HDSS surveys, in order to ascertain cause of death.[9] The VA

Table 3 Sampling strategy by site

		Tanzania	South Africa	Zimbabwe	Malawi	Kenya		Uganda
**Sampling category**		**Kisesa**	**uMkhanyakude**	**Manicaland**	**Karonga**	**Kisumu**	**Rakai**	**Kyamulibwa**
1. Diagnosed but not initiated								
Sampling frame	Groups chosen as either (i) eligible (ii) not eligible (iii) had never undergone investigation				X	X		
	Groups chosen as either (i) contact with HIV care (ii) no contact with HIV care	X	X	X			X	X
Recruitment strategy	Use HDSS and HIV clinic datasets to identify those not in care		X	X	X	X	X	X
	Seeded focus group approach	X						
	Pre-ART consultations at local clinics to identify those already in care	X	X	X	X	X	X	X
	Contact people through outreach, community organisation and HBC groups	X	X	X		X		
	Contact people using details in the linked HDSS and clinic databases				X		X	X
	Snowballing—participants asked to bring a friend			X				
**2. Recently initiated onto ART (within the last 6 months)**								
Sampling frame	1. Timely initiation	X	X	X	X	X	X	X
	2. Urgent initiation	X	X	X	X	X	X	X
Recruitment strategy	Screened for eligibility	X	X	X	X	X	X	X
	Screened for additional characteristics of interest (eg, pregnant women on option B+)	X			X		-	X
	Screened to check individuals are members of the HDSS					X	X	X
	Used pre-ART and ART registers to assess timing of initiation	X	X	X	-	X	X	X
	Used CD4 count records to assess urgency	X	X	X		X	X	X
Interview conduct	Interviews conducted within 1–2 weeks of initiation	X	X	X	X	X	X	X
	Repeat interviews conducted to assess settling in phase	X			X			
**3. Lost to follow-up**								
Sampling frame	Linked HDSS and clinic data to identify LTFU						X	X
	Clinic lists of LTFU used as starting point, then HDSS data to find residency	X			-	X		
	Only clinic records used			X				
Recruitment strategy	Home-based care workers or tracing nurses used to recruit	X			X			
	Clinic-based case managers used to recruit					X		
**4. Been on ART for at least 6 month**								
Sampling frame	Clinic record system used to identify patients who had initiated then screened for treatment duration	X	X	X	X			
	Clinic records for those more than 6 months then linked to HDSS to ensure residency within HDSS					X	X	X
	Patient reports used to ascertain regularity of visits	X	X	X	X	X	X	X
Recruitment strategy	Recruited from clinics	X	X	X	X	X	X	X
**5. Family members of PLHIV who have died**								
Sampling frame	VA database—HIV-related deaths where family members knew the deceased status	X	X	X	X	X	X	X
Recruitment strategy	Consent for being contacted obtained during the VA (preconsent system)	X	X	X	X	X	X	X
**6. Healthcare workers**								
Sampling frame	Purposive sampling of HCT counsellors, home-based care workers, nurses, adherence counsellors and clinicians	X	X	X	X	X	X	X

ART, antiretroviral therapy; HDSS, Health and Demographic Surveillance System; VA, verbal autopsy.

Table 4 Data collection and data processing summary by site

		Tanzania	South Africa	Zimbabwe	Malawi	Kenya	Uganda	
		**Kisesa**	**uMkhanyakude**	**Manicaland**	**Karonga**	**Kisumu**	**Rakai**	**Kyamulibwa**
Timing of data collection	Start date	September 2015	September 2015	February 2016	September 2015	February 2016	September 2015	September 2015
	End date	March 2016	March 2016	May 2016	February 2016	May 2016	March 2016	March 2016
Remuneration	Payment made for participating in the study		X	X			X	X
	Transport reimbursed	X						
	Refreshments provided	X						
Recording of interviews	Data was audio recorded, field notes were taken by interviewer and detailed case notes written post interview	X	X	X	X	X	X	X
Research assistants	Participatory training took place led by lead researcher on the study objectives and procedures	X	X	X	X	X	X	X
	Experienced university graduate researcher conducted interviews	X	X	X		X	X	X
	Experienced secondary school graduate researcher-conducted interviews				X			X
Transcription and translation	Audio recordings were transcribed verbatim and later translated into English	X	X	X				X
	Audio recordings were translated as they were being transcribed				X	X	X	
	Audio recordings were used to make detailed reports that built upon the detailed case notes		X					X
	The research assistants conducted their own transcriptions and translations		X	X	X	X	X	X
	Independent transcribers/translators were used	X			X			X
	Quality checks were made by the study site lead— approximately 50% of transcripts reviewed	X	X	X	X	X	X	X
Case notes	RA used notebooks to capture detailed case notes, these were appended to the transcripts/reports	X	X	X	X	X	X	X
Data storage	Data were stored in secured password-protected locations	X	X	X	X	X	X	X

Table 5 Global themes and contribution of data per site

	Tanzania	South Africa	Zimbabwe	Malawi	Kenya	Uganda	
**Global thematic area and cross cutting themes**	**Kisesa**	**uMkhanyakude**	**Manicaland**	**Karonga**	**Kisumu**	**Rakai**	**Kyamulibwa**
1. Experiences around testing	X	X	X	X	X	X	X
2. Experiences around linkage to ART care	X	X	X	X	X	X	X
3. Experiences affecting retention in care after ART initiation	X	X	X	X	X	X	X
4. Medical pluralism	X	X	X	X	X	X	X
5. Experiences around option B+	X			X	X		X
6. Couples and relationships	X	X		X			
7. Patient—provider interactions	X	X	X	X	X	X	X
8. Experiences leading up to the deaths of PLHIV	X	X	X	X	X	X	X
9. Issues pertaining to stigma	X	X	X	X	X	X	X
10. Critiquing the linearity of the cascade of care	X	X	X	X	X	X	X

ART, antiretroviral therapy; PLHIV, people living with HIV.

interviews are conducted by trained field workers using structured questionnaires to identify physical signs, symptoms and events leading up to a death, followed later by review and interpretation by trained clinicians using a computer-based algorithm to identify the clinical causes of death. The VA tool also includes an unstructured narrative component to document additional information given by the family which is not normally used for clinical interpretation. VA data include information from families on whether a medical diagnosis of HIV/AIDS had been made, and whether the deceased had previous contact with HIV care and treatment services. In order to sample people to interview in this category, recent VA data were used in which HIV-related deaths had been identified. Only those who knew of the deceased's HIV status were sampled.

The sampling for this group included individuals who had

a. died, never accessed HIV care
b. died, entered HIV care but never had ART
c. died, started ART but died after starting treatment.

The target was to interview a minimum of five persons from this category. The VA and/or HDSS data were used to ascertain care-seeking behaviour. A pre-consent system was adopted in all countries in which participants had already consented on the VA to be subsequently contacted. Researchers then made contact with the family to arrange an appointment to conduct an interview at a time or place convenient to them.

Sampling healthcare workers

HIV service providers working in clinics within the HDSS were purposively selected for inclusion into the study. The healthcare workers included HIV counsellors, home-based care workers, nurses, adherence counsellors and clinicians. The number of health workers recruited varied by setting depending on how many clinics were situated in the study area and the number of different types of health workers involved in providing HIV care. The target was to interview approximately 5–10 healthcare workers in each site.

DATA MANAGEMENT AND PROCESSING

All interviews were audio recorded, transcribed and translated into English in all sites except Kyamulibwa and Rakai, where detailed case summaries were prepared after each interview. Data were secured on password-protected computers. Audio files were deleted after transcripts were produced. Data were coded by the study coordinator in each setting, with Nvivo or manually, using a framework analysis approach to identify emerging patterns and relationships between the codes.[10] Table 4 details the data management processes by site.

DEVELOPMENT OF THE ANALYTICAL FRAMEWORK

A second workshop took place in London in September 2015 to (1) review the data that had been collected and discuss key themes emerging from each site's data, (2) refine analysis plans and (3) prepare abstracts for the International AIDS Conference in Durban in July 2016.

The study coordinators from each site were convened, with one site participating by Skype. During the workshop, a broad analytical framework was developed based on the original study protocol and drawing on the different stages of the HIV care continuum (ie, HIV testing, linkage to care, initiation of ART, retention in care). Additional themes that were recurrent across several sites and deemed to be important contributors to bottlenecks in care were also identified (see table 1).

Following on from this workshop, each study coordinator continued this process of categorising key themes emerging from the data. For each topic area, working groups were established to refine the analyses and interpretations of the data. Junior researchers were mentored by more senior researchers within the study team. The initial broad codes conducted at each site were shared with the thematic lead responsible for conducting the next level of analysis for each of the cross-cutting themes (see table 5). Researchers from Karonga and Kyamulibwa shared their thematic analysis in Excel tables while the Manicaland team summarised their analysis in Word. Researchers from Kisesa and Rakai shared NVivo nodes also organised by key themes while those from Kisumu, Kisesa and uMkhanyakude shared full or part transcripts to illustrate aspects of the organising themes. Lead authors conducted the more inductive final analysis. Regular contact took place between all site leaders to ensure consistency and faithfulness to the data and the context from which it arose.

A third meeting among the study coordinators was held during the AIDS conference in Durban in 2016 during which lead authors presented their analyses and draft manuscripts. Each analysis was discussed in detail by the coauthors. Drafts of papers were regularly circulated and discussed between the coauthors. All manuscripts were passed through the appropriate channels within each of the study sites prior to submission.

REFERENCES

1 Kishamawe C, Isingo R, Mtenga B, *et al*. Health & Demographic Surveillance System Profile: The Magu Health and Demographic Surveillance System (Magu HDSS). *Int J Epidemiol* 2015;44:1851–61. http://dx.doi.org/10.1093/ije/dyv188

2 Reniers G, Wamukoya M, Urassa M, *et al.* Data Resource Profile: Network for Analysing Longitudinal Population-based HIV/AIDS data on Africa (ALPHA Network). *Int J Epidemiol* 2016;45:83–93. http://dx.doi.org/10.1093/ije/dyv343

3 Odhiambo FO, Laserson KF, Sewe M, *et al.* Profile: The KEMRI/CDC Health and Demographic Surveillance System—Western Kenya. *Int J Epidemiol* 2012;41:977–87. http://dx.doi.org/10.1093/ije/dys108

4 Church K, Machiyama K, Todd J, *et al.* Identifying gaps in HIV service delivery across the diagnosis-to-treatment cascade: Findings from health facility surveys in six sub-Saharan countries. *J Int AIDS Soc* 2017; 20:1–14. http://www.jiasociety.org/index.php/jias/article/view/21188

5 Wringe A, Renju J, Seeley J, *et al.* Bottlenecks to HIV care and treatment in sub-Saharan Africa: A multi-country qualitative study. *Sex Transm Infect.* In press.

6 Busza J, Zaba B, Urassa M. The 'seeded' focus group: a strategy to recruit HIV+ community members into treatment research. *Sex Transm Infect* 2009;85:212–5. http://sti.bmj.com/content/85/3/212.abstract

7 Floyd S, Marston M, Baisley K, *et al.* The effect of antiretroviral therapy provision on all-cause, AIDS and non-AIDS mortality at the population level – a comparative analysis of data from four settings in Southern and East Africa. *Trop Med Int Heal* 2012;17:e84–e93. doi:10.1111/j.1365-3156.2012.03032.x

8 Church K, Kiweewa F, Dasgupta A, *et al.* A comparative analysis of national HIV policies in six African countries with generalized epidemics. *Research* 2015;:457–67. doi:10.2471/BLT.14.147215

9 McCormick TH, Li Z, Calvert C, *et al.* Probabilistic cause-of-death assignment using verbal autopsies. *J Am Stat Assoc* 2014;1459. doi:10.1080/01621459.2016.1152191

10 Attridge-Stirling J. Thematic networks: an analytic tool for qualitative research. *Qual Res* 2001;1:385–405. doi:10.1177/1468794107085301
